# Construction and Preparation of Three Recombinant Adenoviruses Expressing Truncated NS3 and Core Genes of Hepatitis C Virus for Vaccine Purposes

**DOI:** 10.5812/hepatmon.6130

**Published:** 2012-08-14

**Authors:** Seyed Younes Hosseini, Farzaneh Sabahi, Seyed Mohammad Moazzeni, Mohammad Hossein Modarressi, Mehdi Saberi Firoozi, Mehrdad Ravanshad

**Affiliations:** 1 Department of Virology, Tarbiat Modares University, Tehran, IR Iran; 2 Gastroentero -Hepatology Research Center, Shiraz University of Medical Sciences, Shiraz, IR Iran; 3 Department of Immunology, Tarbiat Modares University, Tehran, IR Iran; 4 Department of Medical Genetic, Tehran University of Medical Sciences, Tehran, IR Iran; 5 Digestive Disease Research Center, Tehran University of Medical Sciences, Tehran, IR Iran

**Keywords:** Vaccines, Genetic Vector, Genes

## Abstract

**Background:**

In spite of dozens of clinical trials to establish effective therapeutic and/or preventive vaccine to resolve HCV infection, no real vaccine has been proved to date. Genetic vaccines based on replication-defective adenoviruses have proved to elicit strong and long lasting T-cell responses against a number of viral antigens and are even currently being used for vaccine trials in humans. According to the controversy in the immune modulatory effects of both core and NS3 full length genes, it seemed more practical to employ some parts of these HCV proteins for vaccine design.

**Objectives:**

To generate recombinant Adenoviral vectors containing new overlapping-truncated region of NS3 gene or both the N- and C-terminal deleted parts of core gene, as well as a fusion fragment derived from both of them.

**Materials and Methods:**

The corresponding transfer vectors expressing truncated fragments of core, NS3 or a fusion fragment of both genes were prepared. The integrity and sequence of the transfer vectors were confirmed, and followed by experiments involving homologous recombination between them and the adenovirus backbone plasmid in the bacterial host. Recombinant Ad-pNS3, Ad-pCore and Ad-pNS3pCore viruses were prepared by transfection of these new recombined constructs into 293 packaging cell lines. The virus titer was then calculated by an immunohistochemistry based method. The RT-PCR, Real-Time PCR and western blotting were used to evaluate gene expression by all recombinant constructs. The production of complete virion particles was evaluated by detailed electron microscopy in addition to the appearance of typical cytopathic effects (CPE) and GFP expression patterns in 293 cells. The RT-PCR and GFP detection were employed to monitor the integrity as well as infectivity potency of the viral particles in Hep-G2 cells.

**Results:**

RT-PCR, Real-Time PCR or western blotting confirmed expression of truncated fragment of NS3, core or a fusion fragment of theirs by newly constructed Ad-pNS3, Ad-pCore, Ad- pNS3pCore particles. Electron microscopy, which revealed many adenovirus-like particles and characteristics of CPE in infected cells in addition to GFP detection, confirmed the infectivity, potency and integrity of recombinant adenoviral particles.

**Conclusions:**

These adenoviruses expressing novel fragments of NS3 and core genes may be suitable tools to overcome shortcomings associated with full gene expression in the setting of HCV vaccine therapy.

## 1. Background

In spite of dozens of clinical trials to establish effective therapeutic and/or preventive vaccine to resolve HCV infection, no real vaccine has been proved to date (1). Cellular immunity is essential for spontaneous resolution of acute hepatitis C and long-term protection from persistent infection (2, 3).Genetic vaccines based on replication-defective adenoviruses have proved to elicit strong and long lasting T-cell responses against a number of viral antigens, and even currently, are being used for vaccine trials in humans (4, 5,6). Furthermore, employing this vector is a continually growing field for prophylactic or therapeutic purposes in HCV infection (6, 7, 8, 9, 10). Therefore the use of recombinant adenoviral system to stimulate and expand HCV-specific CTL response is an attractive strategy and may be useful in clinical settings (8). This system would result in the intracellular expression of recombinant HCV proteins, which allows endogenously synthesized antigens to be processed via MHC class I pathway and presented on the cell surface, similar to those of natural infection (6, 8, 9). The core and NS3 of HCV have been utilized as important immune genes in various vaccine development studies (11, 12,13). Non-structural protein 3 (NS3) is a protein with important protease and RNA helicase activities. The cellular immune response against it determines the viral persistence outcome (6, 14). Its importance in determining the viral persistence outcome, essential enzymatic activity, and suitable results that were achieved in vaccine studies, make NS3 protein an ideal option for the purpose of anti-HCV vaccine design (15, 16, 17). On the other hand, there are a couple of controversial reports which indicate the immunosuppressive effects of full NS3 protein on antigen presenting cells (APCs) (18, 19). It seems that the new vaccine design based on removing the enzymatic activity of NS3 or even the selection of truncated fragments would be able to boost immune induction by avoiding the protease function of nS3 in APCs and other immune cells (18, 20, 21). At present, protease blocked NS3 employed in an inactivated yeast system is in phase II of clinical evaluation, indicating the benefit of removing this enzymatic activity (20). HCV core protein, as the most conserved antigen among different HCV genotypes, has been employed extensively for induction of cellular immunity in animal models as well as the human models (10, 14, 22, 23). Although the core antigen was employed in clinical trials, some publications indicated the autoimmune property of both C and N-terminal domain sequences of core protein that may be harmful for human vaccine purposes (24). Fur- thermore, other studies have indicated that expression of full core protein leads to immune suppression/modu- lation in animal models (25, 26, 27). In the laboratory, three new recombinant Adenoviruses expressing truncated fragments of core and/or NS3 proteins were prepared to avoid immune modulatory/suppressive effects of full length genes (24, 28, 29). According to the controversy in the immune modulatory effects of both core and NS3 full length genes, it seemed more practical to employ some parts of these HCV proteins for vaccine design, considering their proven roles in viral clearance and their conserved sequence.

## 2. Objectives

Employing the adenoviral vectors expressing specific parts of HCV proteins may overcome the present short- comings related to full-length protein expression, especially in APCs. It ultimately helps to overcome some of the defaults related to standard therapy.

## 3. Materials and Methods

### 3.1. Reagents, Vectors and Cell Lines

The T4 ligase and restriction enzymes were purchased from New England Biolab (USA). Plasmid and DNA isolation/purification kit were purchased from Bioneer (S. Korea), InsTAclone™ PCR Cloning kit, BstxI, and Pfu DNA polymerase, in addition to other routine molecular reagents were purchased from Fermentas Company (Thermo). Lipofectamin ^TM^ 2000 from Invitrogen (USA), culture media and other culture related materials were purchased from Gibco (USA), and were stored in the recommended milieus. All antibodies were purchased from Abcam Company and were stored at -70°C for long-term storage. Clontech^TM^Adeno rapid titer kit was purchased from Clontech Company for titration of viral stocks. Adenovator^TM^ expression system and pAdenovator-CMV5-IRES-EGFP shuttle plasmid were purchased from Qbiogene Corporation. The Low-passage HEK 293 and Hep-G2 cell lines were provided by the National Cell Bank of Iran, and were grown in Dulbecco’s modified Eagle’s medium (DMEM) supplemented with 100 IU penicillin ml^−1^, 100mg streptomycin ml^−1^, 10% (v/v) fetal bovine serum, 20mM HEPES and non-essential amino acids.

### 3.2. Construction of Various Shuttle Plasmids

**Figure 1:**
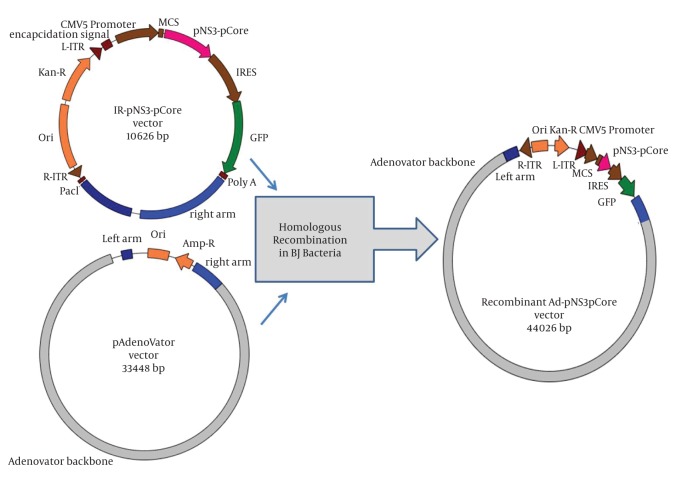
Schematic Representation of Homologous Recombination Between Constructed Shuttle Vector (IR-pNS3-pCore) and pAdenoVator in BJ Bacterial Host Linearized shuttle plasmid together with supercoiled pAdenovator co-transformed into bacterial host to lead recombination. Finally, the created recombinant Ad-pNS3-pCore vector contains all shuttle plasmid elements rather than adenovirus backbone. Recombination lead to replacement of all auxiliary genetic elements inside the pAdenoVator with those of linearized shuttle plasmid as described in the text.

Amplification of new partial core (aa 50-160), new overlapping region of NS3 gene covering protease/helicase domains and a fusion of both truncated fragments from HCV genotype 1a were previously described (30, 31). In brief, full core and partial NS3 sequences were propagated from the serum of a HCV patient by RT-PCR. The resulting extracted amplicons were first cloned into the pTZ57R/T cloning vector. After primary confirmation of the prepared core gene, to amplify partial core harboring amino acid sequence 50-160 which is an N and C-terminal deleted sequence, a pair of primers containing Nde-I restriction site was exploited on a pTZ57R/T vector containing full core gene. The resultant amplicon of core gene was inserted into the TA-pNS3 construct at NdeI site, making a new fusion sequence of pNS3 and pCore. Finally, the gene fragments were inserted into pAdenovator-CMV5-IRES-EGFP vector as shuttle vector. The transfer/shuttle vectors contained partial NS3 or partial core and fusion of both genes designated as IR-pNS3, IRpCore, and IR-pNS3-pCore (Figure 1) respectively. Phylogenetic analysis of sequences was also used to confirm true cloning of the desired genotype as described previously (30, 31).

### 3.3. Construction of Recombinant Adenoviral Vectors

To generate recombinant adenoviral plasmid expressing genes, each high quality PmeI linearized IR-pCore, IR-pNS3, and IR-pNS3pCore transfer vector together with supercoiled pAdenovator Δ E1/E3 containing Ad5 genome were co-transformed into electro competent E. coli BJ5183 to prompt homologous recombination between them (Figure 1) as described previously, followed by 16-18 hr incubation on LB medium containing kanamycin (32, 33). The proper Adenovirus recombinants which form colonies were selected by two different colony-PCRs using gene specific primers and a new pair of primers, Fib-1 and Fib-2, which were designed to amplify a 1700 bp fragment of the fiber region (Table 1). Restriction analysis with PacI and BstxI enzymes was also exploited to verify the suitable recombinant constructs as the final step of confirmation. These newly constructed recombinant adenovectors containing pCore, pNS3 and pNS3pCore fragments, designated as Ad-pCore, Ad-pNS3 and Ad-pNS3pCore, were digested by the PacI enzyme and then purified by traditional ethanol precipitation method.

**Table 1:** The Sequences of the Primers Used in This Study a in The Case of Hepatitis C Virus Genes a

	**Function of Primer**	**Position**	**Sequence 5’-3’**
Q-core 1	Quantification of partial core	517-536	GAGGTAGACGTCAGCCTATC
Q-core 2	Quantification of partial core	686-704	TTACCCAAATTACGCGACCT
Fib-1	Detection of Adeno Fiber	-	CCACTATCTTCATGTTGTTGCAG
Fib-2	Detection of Adeno Fiber	-	AGAATCGTTTGTGTTATGTTTCAACGTG

^a^The primers sequences and positions are based on HCV complete genome reference sequence strain H77 (GenBank accession number AF009606)

### 3.4. Virus Preparation, Purification and Quantification

To produce viruses, first separate 25cm^2^ flasks of low passage HEK-293 cells were transfected with a mixture of each PacI digested Adenoviruses/Lipofectamine 2000^TM^ as recommended by the company, then the recombinant Adenoviruses were allowed to produce and propagate efficiently for at least six days past transfection (33). HEK-293 cells were transfected with linear Adenovirus constructs while growing in logarithmic phase. They were fed by replacing 2 ml DMEM complete medium and examined for the evidence of cytopathic effects or GFP expression every two-three days. The cells were harvested when GFP green signals turned into distinct shaped foci under microscopy. For virus collection the cells were precipitated by centrifuge and then lysed by three consecutive freeze-thawing cycles in ice-methanol bath. The supernatant containing the released viral particles was harvested after a low speed centrifuge. To generate high titer viral stocks, the HEK 293 cells were re-infected three-four times sequentially by the cell lysate supernatant, then the final reinfection and harvesting processes were repeated in more than 30 × 175 cm^2^ flasks to achieve enough supernatant containing the recombinant virus. For virus purification the final harvested cells were lysed in PBS-EDTA by three consecutive freeze-thawing cycles, and the viruses were collected from supernatants after low speed centrifugation. After passing the crude supernatant through a 0.4 μm syringe filter, they were purified by two steps ultracentrifuge on cesium chloride gradient. The final band was extracted and applied into an Amicon ultra-15^TM^ (Milipore company) desalting column to remove inadvertent materials from the viral solution. The titer of the viral stocks was first estimated as viral particles/ml by measuring the optical density (OD) of the purified viral genomes using spectrophotometery. The titers of complete infectious viruses were then calculated as Infectious Unit/ml (IU/ml) by commercially available Clontech^TM^Adeno rapid titer kit under standard conditions. Then 100 ul of serially diluted viral stocks were left 1.5 hr on 10^5^ 293 cells per well in a 96-well plate as vector adsorption period, and complete medium was then added up to 200 ul following 48 hours incubation time. The final detection of viral propagation in 293 cells by immunohistochemistry method was accomplished by company recommendation and, at the end, by introducing the results into the formula; the dose of infectious viruses per volume was calculated as described previously (34). Final stock was stored in PBS-EDTA containing 10% glycerol buffer at -80°C. To evaluate the viral genome, the viruses were isolated from 293 cell lysates, and viral DNA was extracted as reported previously (37).

### 3.5. Identification and Confirmation of Adenovirus Vectors

Screening of virus propagation was assessed by detection of GFP at different time intervals. After visualization of GFP expression as screening route, the presence of recombinant viral genome was evaluated byPCR for fiber gene by specific primers (Table 1) on HEK-293 lysate after the second round of reinfection. Fluorescent images were captured at 490 nm using a Nikon Eclipse E2000 microscope and viral DNA was extracted based on proteinase K treatment followed by boiling as previously described (35). For ultra-structural analysis of the viral particles, electron microscopy was employed to confirm virus assembly and integrity. To accomplish that, under GLP conditions concentrated vector stock was thawed, diluted (1:20) with pure water, then evaluated after routine preparation of grids. After negative staining in phosphor tungstic acid, the grids were evaluated and photomicrographed with a Zeiss electron microscope with a final magnification of 120,000 times.

### 3.6. Infectivity Test

HEK-293 and Hep-G2 cell lines were employed to evaluate the potency of adenovirus infectivity. Cells having 80-90% confluency were infected with Ad-pCore, Ad-pNS3 or Ad-pNS3-pCore viruses at 10-20 MOI (multiplicity of infection). The medium was changed after two hours of absorption followed by confirmation of GFP expression by fluorescent microscopy as well as flow cytometery analysis after 24-48 hours. The characteristic cytopathic effect of Adenovirus also served to monitor transduction rather than infectivity potency in HEK-293 cells.

### 3.7. Analyses of Gene Expression

The HEK-293 or Hep-G2 cells were cultured in six well plates and infected with all adenovirus vectors including Ad-pNS3, Ad-pCore, and Ad-pNS3pCore, in addition to Adeno-GFP virion as negative control to analyze the gene expression. These cells were harvested up to 48 hours after transfection. After a brief screening by GFP expression under fluorescent microscopy, genomic RNA was extracted using total RNA extraction kit following DNase-I treatment, while RT-PCR was done with the corresponding specific primers mentioned here in (Table 1) or previously mentioned (31). Also, an in-house Real-time PCR based on cyber green was exploited to evaluate the partial core gene expression by Ad-pCore and Ad-pNS3pCore using BioRad C1000^TM^ thermal cycler. The Q-core primers were designed for core gene quantification (Table 1) so that nearly a 200 bp amplicon was made and the resultant cyber green signal was evaluated after 40 cycles of amplification. Reaction cycles included denaturation cycle at 94°C for 30 s, annealing at 60°C for 50 s, and extension at 72°C for 10 s. The CT value was plotted against the serial dilution of a full core gene containing plasmid to generate a standard curve that can be used to quantify unknown samples by CT value. For SDS–PAGE analysis, cell lysate samples were mixed in SDS–gel sample buffer, heated at 100°C, then separated in 12% SDS–polyacrylamide gels. The Western blotting was performed as described earlier (36) using commercial rabbit polyclonal antibodies for NS3 protein. After blotting the proteins onto nitrocellulose membrane and blocking by BSA, they were treated by diluted antibodies, then washed thoroughly and incubated by TBS containing HRP-labeled goat anti-rabbit IgG (Sigma–Aldrich). After being washed, the membrane was developed by employing the ECL plus ^TM^ blotting system from Amersham Company.

## 4. Results

### 4.1. Shuttle Plasmids Preparation and Analysis of Gene Sequences

As described in the previous report partial NS3, partial core or fusion of both genes were cloned step by step respectively into IR-pNS3, IR-pCore or IR-pNS3-pCore Transfer/shuttle vectors under the control of CMV5 promoter (Figure 1). The sequence of the full core gene showed more than 99% similarity with those of other 1a sequences using multiple alignment survey. However, the NS3 gene from the Iranian patient had more differences in gene sequence, albeit a high homology in amino acid sequence (unpublished data) in comparison to other reference sequences. Evaluation of the amplified NS3 sequence by phylogenetic analysis strongly confirmed that it belonged to 1a genotype as was previously described in detail (30).

### 4.2. Construction of Recombinant Adenoviral Vectors

**Figure 2:**
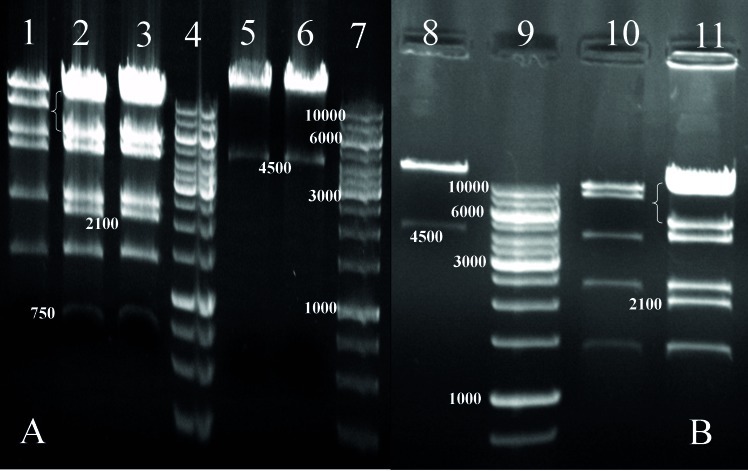
Digestion Analysis of Recombinant Adenovirus Constructs by BstXI and PacI (A) Restriction Pattern for recombinant Ad-pCore construct, 1: negative control including pAdenovator Ad5 genome digested with BstXI and appearance of special 6 band pattern. 2 and 3: Two true colonies of Ad-pCore construct digested with BstXI. Appearance of a nearly 700 bp band as evidence of Core gene insertion, presence of a 2.1 kb fragment as recombination clue as well as disappearance of original 8 kb band(bracket) in recombinant construct approved vector, 4: DNA ladder 1kb Fermentas cop., Cat. Number: SM1163, 5 and 6: true colonies of Ad-pCore constructs digested with PacI. Appearance of a 4.5 kb and big fragment (upper than 10kb) was a sign of gene insertion and vector integrity, 7: DNA ladder, (B) Restriction Pattern for recombinant Ad-pNS3 construct 8: digestion of Ad-pNS3 construct with PacI showed the appearance of a 4.5 kb and a big fragment (upper than 10kb) which implied the vector integrity, 9: ladder, 10: a false/ wrong colony digested with BstXI, 11: digestion of right Ad-pNS3 construct by BstXI, detection of 2.1 kb fragment and disappearance of original 8 kb (bracket) band was evidence of gene insertion as well as construct integrity.

The IR-pCore, IR-pNS3 and IR-pNS3pCore transfer vectors were separately transformed with supercoiled pAdenovator Δ E1/E3 into BJ bacterial host and, consequently, by homologous recombination, new Adenovirus recombinant plasmids were created as shown in Figure 1. As a confirmatory step to evaluate recombinant Ad-pCore, Ad-pNS3 and Ad-pNS3pCore integrity, these constructs were treated and analyzed with BstXI and PacI restriction enzymes according to companies’ guidelines. Based on the guidelines, digestion of an Adenovirus containing gene of insert with BstXI enzyme should appear in a six band pattern, especially a 2.1 kb fragment as evidence of gene insertion after a long run on 1% agarose gel. In the case of Ad-pNS3, the result was the same as expected but for Ad-pCore and Ad-pNS3pCore, because of the presence of the newly emerged BstXI restriction site at original core gene, Other bands around 700 and 900 base pairs were detected, which confirmed gene insertions as shown in Figure 2. Digestion analysis by PacI also showed the presence of a 4.5 kb fragment below a big plasmid backbone fragment as further evidence for integrity and also right insertion of genes into the construct (Figure 2).

### 4.3. Identification and Quantification of Recombinant Viral Particles

**Figure 3:**
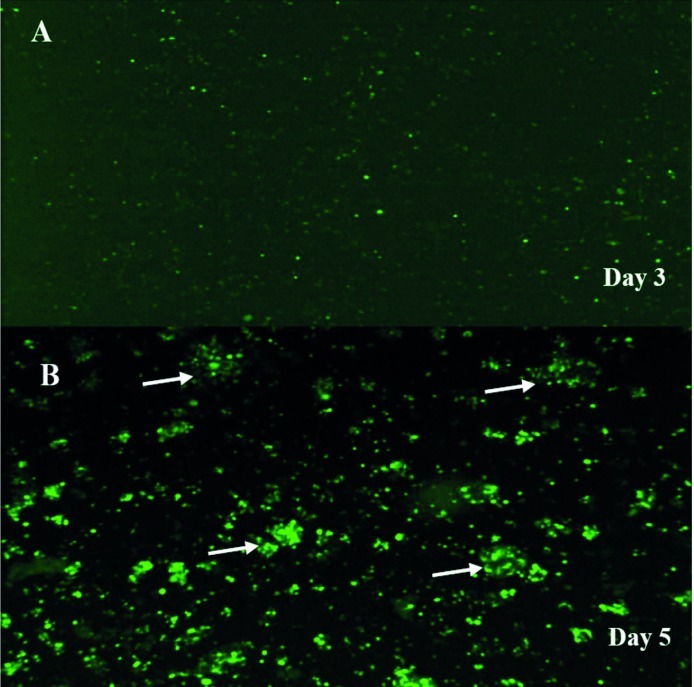
Adenovirus-Producing Foci as a Sign of Virus Propagation PacI-digested Ad-pNS3 construct was transfected into 293 cells and GFP expression was visualized by fluorescence microscopy thereafter, (A). On day three after transfection of 293 cells, single cells expressing GFP were representative of virus gene expression and virus formation, (B). After five-six days post-transfection, Comet-like adenovirus-producing foci became apparent as marked by arrow head. This phenomenon is an indicator of propagation in addition to spreading the infectious virus through neighboring cells. No such foci were observed in the cells transfected with circular (i.e., not PacI-digested) Ad-pNS3.

**Figure 4:**
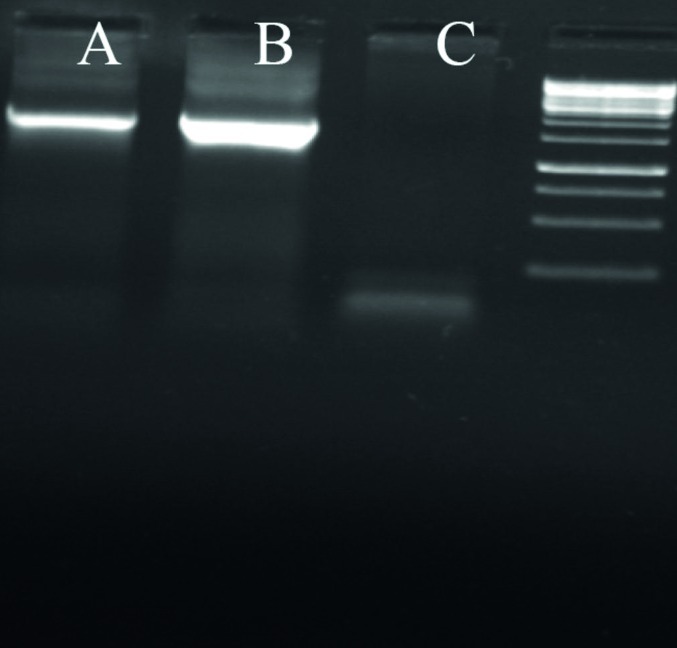
Fiber Amplification as a Confirmatory Step of Recombinant Virus Presence After two step of reinfection, supernatant or lysed cells undergo a 25 cycle PCR reaction to amplify 1700 bp from fiber gene as virus production sign. As shown, no significant difference was seen between the sample processed by standard lyses method or supernatant medium. From left to right, A) sample from supernatant without lyses B) sample prepared by standard lyses method C) 293 negative control.

**Figure 5:**
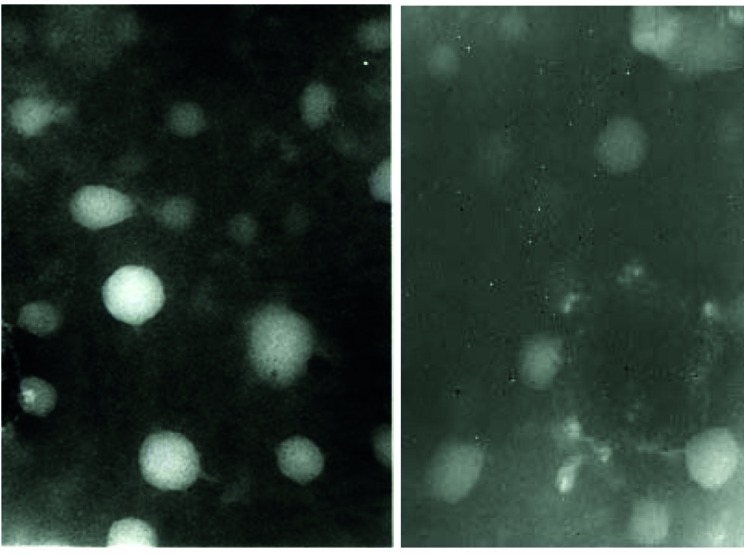
Two distinct Electron Microscopy Images Showed Many Icosahedral Adenoviral Particles as Single or Cluster Forms with Hardly Visible Fiber Projections Surrounded by Some Defective Particles

In order to screen the formation and amplification of first viral particles, cultured cells were evaluated to detect GFP emission every two days. During the first three-four days after transfection of linear Adenovirus constructs into cells, there was a GFP pattern such as the one observed in traditional transfection (Figure 3). After five-six days, as a consequence of spreading the released viruses through neighboring cells in culture dishes the pattern of foci appeared as a clue for reproductive amplification of recombinant viral particles as shown in Figure 3. Special view of comet-like fluorescence or a focus forming pattern under fluorescent microscopy at days five-seven post transfection served as an important clue for vector production as explained before (37).To evaluate the presence of the viral genome as a direct sign of virus production, PCR test for fiber of adenoviral vector was developed. The PCR with fiber specific primers on cell lysate showed a sharp 1700 bp fragment, indicating the propagation of Adenovirus particles after two cycles of reinfection with 293 cell line. Interestingly, no significant difference was seen between the PCR results of the samples prepared from the cells by proteinase K lysis method or untreated culture supernatant (Figure 4). Electron microscopy was employed for detailed assessment of the production of viral particles in all virus batches. Electron microscopy revealed many Adenoviruses like particles in clusters or single harboring faint fibers projected from the surroundings of the particles (Figure 5). Among these complete Adenoviruses, many defective particles were also detected with similar shape but smaller size at the background. After viral purification, titer of recombinant viral stocks was evaluated as both particle/ml and Infectious particle/ml as described under the mentioned methods. The results indicated that the ratio of infectious particles to total particle counts was less than 1/200 in all virus batches. The real titers of these batches were calculated to be around 10^9^ infectious units/ml.

### 4.4. Infectivity Test

**Figure 6:**
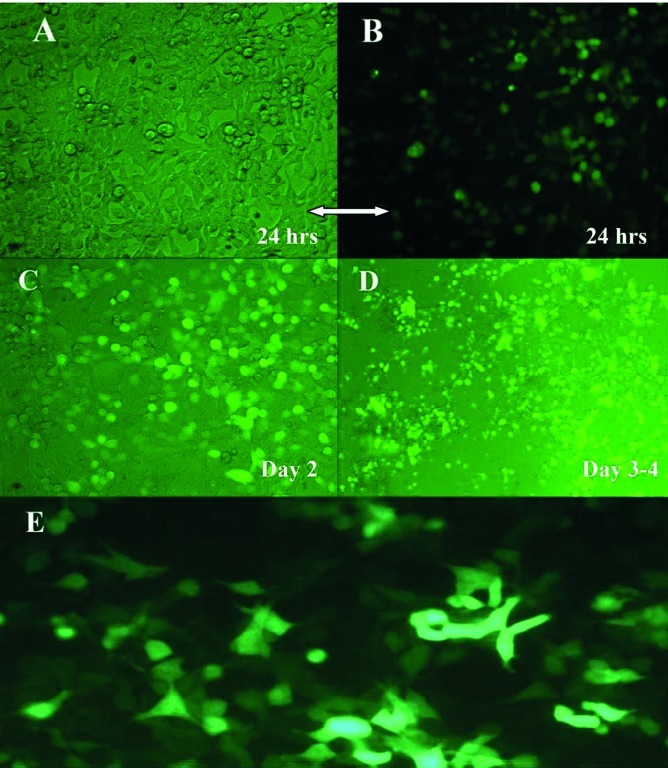
Concomitant of GFP Expression and Cytopathic Effect at Different Times in Infectivity Test (A-D) Infection of 293 permissive cells by Ad-pNS3, expression of GFP and CPE appearance. Thereafter, (A and B) 24 hours after infection of 293 cells and appearance of rounded semi-adherent cells in slide A that appeared as sharp green by fluorescent microscopy in slide B, (C) Overlay image of day two after infection of 293 cells and increased amount of roundedgreen cells on the polygonal cells in null color, (D) Day three-four post infection and devised cells caused a big empty space. Nearly all the cells had signs of GFP expression inside, (E) Hep-G2 cells after infection by AdenopNS3pCore. After two days past infection at MOI 15, fluorescent microscopy showed bright green in more than 70 percent of the cells as a sign of GFP expression, virus infectivity and possibly expression of gene of interest, pNSpCore fused fragment

Infection of HEK-293 or Hep-G2 cell line with prepared adenoviral vectors showed expression of GFP protein under fluorescent microscopy after 48 hours past infection (Figure 6). To confirm fluorescent microscopy results in detail, Flow cytometery analysis also showed expression of GFP in more than 80% of live population in assessed samples. After infection of HEK-293 cells, adenovirus cytopathic effect (CPE) started to appear 24 hours post infection. As time passed, rounded and swelled cells began to detach from the plate and float in supernatant. After that, on days two-four the CPE grew more by increasing the number of rounded cells, which then fully detached and resulted in creating empty spaces in culture dishes as shown in Figure 6.

### 4.5. Analysis of Gene Expression

**Figure 7:**
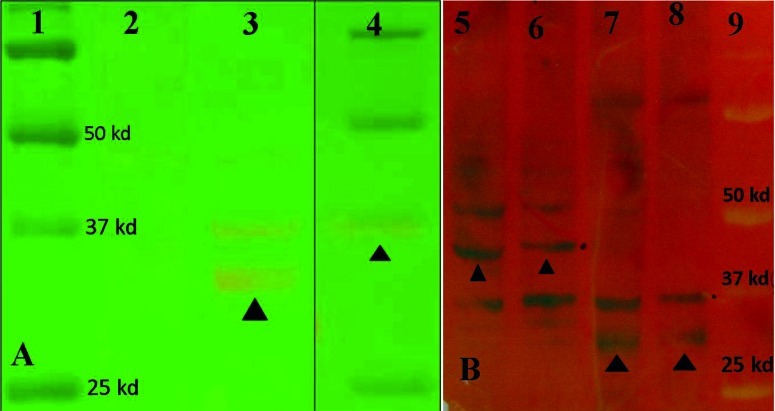
Results of Western Blotting Analysis of pNS3 and pNS3pCore proteins Expression by Ad-pNS3 and Ad-pNS3pCore viruses in 293 Cells (A): Western blot analysis of pNS3 protein in 293 cells. 1- Protein Marker 2- Uninfected 293 cells as control negative, 3- Ad-pNS3 virus expressing pNS3 protein with around 32 kd weight (filled triangle), 4- A mix of Control positive and protein marker together, helicase part of NS3 purified protein connected to a special Tag that had been prepared in (J. Lasarte Lab, CIMA, Spain) with estimated 35 kd weight showed by filled triangle standed on 37 kd standard. Prestained Protein marker Cat. Number: 161-0374 (BioRad cop.) (B): Western blot analysis of pNS3 and pNS3pCore proteins expression in 293 cells by Ad-pNS3 and Ad-pNS3pCore virus. 5 and 6- Ad-pNS3pCore virus expressing pNS3pCore protein with estimated 45 kd weight (triangles). 7 and 8- Ad-pNS3 virus expressing pNS3 protein with around 32 kd weight (triangles). 9- Pre-stained Protein marker Cat. Number: 161-0374 (BioRad cop.)

After production of virus stocks, gene expression was analyzed by RT-PCR, Q-PCR and western blot. The RT-PCR results indicated suitable expression of all genes after viral transduction into Hep-G2 or 293 and evaluation of total extracted RNA. Western blot analysis also showed suitable expression at protein level in transduced cells. Sharp protein bands were seen after western blot analysis of both recombinant Ad-pNS3 and Ad-pNS3pCore viruses at expected positions on paper (Figure 7). The putative molecular weight of two new proteins by SDS–PAGE was estimated to be around 32 kDa for pNS3 and approximately 45 kDa for pNS3pCore fragment (410 a.a), according to 70 kDa size for the full length NS3 sequence and also another similar study (20). The Real-time PCR for core gene showed good expression of pCore and pNS3pCore by Adenoviruses in Hep-G2 cells in comparison with Adeno-GFP as negative control. Also, the GFP detection in accordance with the mRNA presence was a suitable clue to express the protein due to navigation of both GFP and gene of interest by one strong CMV promoter.

## 5. Discussion

In recent years dozens of HCV candidate vaccines have been developed for both prophylactic and therapeutic goals, many of which are classified as Genetic vaccines. So far, modified Vaccine virus, DNA plasmid, inactivated yeast and recently recombinant Adenovirus encoding HCV genes have been developed as HCV genetic vaccine candidates (12, 13). The clinical trial results in patients have shown hopeful prospects for using them alone or in combination with standard therapy. GI5005, yeast expressing novel fusion sequence of NS3-core is a well-tolerated therapeutic vaccine which, in combination with standard therapy obtains suitable results in chronic HCV patients (12, 38). Genetic vaccines based on replication-defective adenoviruses have proved their ability to elicit strong and long lasting T-cell response against a number of viral antigens and are currently used for vaccine trials in humans (4-6, 39). The Adenovirus vector expressing partial or full length of core and NS3 genes from HCV have been exploited for vaccine purposes because of the suitable immunogenicity and high homology of these genes among genotypes (6, 8, 10, 20, 24, 39, 40). Among new promising HCV vaccine candidates, immunogenicity of rare serotype Adenoviruses expressing NS3-NS5 vaccine are being evaluated in human volunteers. The success of these recombinant adenovirus vaccines will provide new hope for the development of effective therapeutic vaccines against HCV (41). NS3 is a bi-functional protein with protease and helicase activities (14). So the NS3 primers were designed in such a way that a major part of the protease enzyme could be amplified rather than the first 1/3 of helicase domain as similarly reported elsewhere (20). Theoretically deleting the first 22 amino acids of protease enzyme reduces or even abolishes enzymatic activity (42). This kind of design may reduce the unwanted protease/helicase activity of NS3 protein, especially in APCs in order to boost immune responses like those evaluated in GI-5005, a yeast expressing fusion of NS3-core (43, 44). Here, an adenovirus expressing partial NS3 gene without N-terminal part was constructed, while harboring was a major part of the protease domain and also a piece of helicase domain. The HCV core protein showed immune modulatory activity and autoimmune induction properties in antigen presenting cells (APCs) while being evaluated as a vaccine candidate (14, 26). In this project, a new Adenovirus expressing a truncated core sequence without N- and C-terminal was prepared to avoid autoimmunity and/or immune modulatory effect of the core full gene sequence (24, 28).The sequencing results showed a very high similarity between the amplified core genes with the other 1a reference sequences as expected. The appearance of a new BstXI restriction site among the new pCore genes made it easier to discriminate between recombined true construct and the other forms by detecting a nearly 700-900 bp fragment after digestion.

The alignment and sequencing results were a little different for partial NS3 amplified target, as expected. More nucleotide differences were detected inside the pNS3 sequence in comparison with those of reference sequences. Virus dosing, which was done by both physical (quantification of genome by OD measurement) and biological assays (rapid commercial method for measuring the infectious unit) showed that the ratio of defective to infective virus particles was less than 200; suitable for further experiments. Infectivity test on 293 and Hep-G2 cells also showed suitable results using fluorescent microscopy and flow cytometery as biological markers for vector integrity. Here, preparation of three new recombinant Adenoviruses expressing truncated forms of the core or middle part of NS3 as well as a fusion form of both fragments was described. The origin of the genes was from an Iranian patient with genotype 1a. Theoretically our Adenovirus vectors should be able to induce suitable cellular immune responses and would help us avoid some shortcomings related to expression of full genes on APCs rather than autoimmunity induction.
